# STAT3 activation in circulating myeloid-derived cells contributes to retinal microvascular dysfunction in diabetes

**DOI:** 10.1186/s12974-019-1533-1

**Published:** 2019-07-08

**Authors:** Mei Chen, Gideon Obasanmi, David Armstrong, Nuala-Jane Lavery, Adrien Kissenpfennig, Noemi Lois, Heping Xu

**Affiliations:** 1 Wellcome-Wolfson Institute for Experimental Medicine, Queen’s University Belfast, Belfast, Northern Ireland UK; 2 Current address: Biomedical Sciences Research Institute, Ulster University, Coleraine, UK

**Keywords:** Diabetic retinopathy, SOCS3, Monocytes, Inflammation

## Abstract

**Background:**

Leukostasis is a key patho-physiological event responsible for capillary occlusion in diabetic retinopathy. Circulating monocytes are the main cell type entrapped in retinal vessels in diabetes. In this study, we investigated the role of the signal transducer and activator of transcription 3 (STAT3) pathway in diabetes-induced immune cell activation and its contribution to retinal microvascular degeneration.

**Methods:**

Forty-one patients with type 1 diabetes (T1D) [mild non-proliferative diabetic retinopathy (mNPDR) (*n* = 13), active proliferative DR (aPDR) (*n* = 14), inactive PDR (iPDR) (*n* = 14)] and 13 age- and gender-matched healthy controls were recruited to the study. C57BL/6 J WT mice, SOCS3^fl/fl^ and LysM^Cre/+^SOCS3^fl/fl^ mice were rendered diabetic by Streptozotocin injection. The expression of the phosphorylated human and mouse STAT3 (pSTAT3), mouse LFA-1, CD62L, CD11b and MHC-II in circulating immune cells was evaluated by flow cytometry. The expression of suppressor of cytokine signalling 3 (SOCS3) was examined by real-time RT-PCR. Mouse plasma levels of cytokines were measured by Cytometric Beads Array assay. Retinal leukostasis was examined following FITC-Concanavalin A perfusion and acellular capillary was examined following Isolectin B4 and Collagen IV staining.

**Results:**

Compared to healthy controls, the expression of pSTAT3 in circulating leukocytes was statistically significantly higher in mNPDR but not aPDR and was negatively correlated with diabetes duration. The expression of pSTAT3 and its inhibitor SOCS3 was also significantly increased in leukocytes from diabetic mice. Diabetic mice had higher plasma levels of IL6 and CCL2 compared with control mice. LysM^Cre/+^SOCS3^fl/fl^ mice and SOCS3^fl/fl^ mice developed comparative levels of diabetes, but leukocyte activation, retinal leukostasis and number of acellular capillaries were statistically significantly increased in LysM^Cre/+^SOCS3^fl/fl^ diabetic mice.

**Conclusion:**

STAT3 activation in circulating immune cells appears to contribute to retinal microvascular degeneration and may be involved in DR initiation in T1D.

**Electronic supplementary material:**

The online version of this article (10.1186/s12974-019-1533-1) contains supplementary material, which is available to authorized users.

## Background

Diabetic microvascular damage affects the eye (retinopathy), kidney (nephropathy) and the nervous system (neuropathy). Although hyperglycemia is the ultimate cause of various diabetic complications, increasing evidence suggests the involvement of inflammation in the initiation and progression of microvascular complications, including diabetic retinopathy (DR) [1, 2]. Inflammation is an adaptive response of the host to endogenous and exogenous insults [3–5]. During diabetes, hyperglycaemia is a chronic insult to circulating immune cells and vascular endothelial cells. Furthermore, various intermediate metabolic products such as advanced glycation end-products (AGEs) and advanced lipoxidation end-products (ALE) also constitute insults to tissue cells [6–8]. Vascular endothelial damage is a main pathology in DR and microaneurysms, a characteristic feature of DR, is believed to be the consequence of endothelial and pericyte cell death [1].

Previous studies have shown that increased leukocyte-endothelial interaction (leukostasis) is a critical step in diabetes-induced retinal microvascular degeneration [9–11]. Leukostasis is considered as a low-grade intravascular inflammatory response [9] that occurs from the early stages of diabetes, well before any detectable clinical manifestations have developed [9, 10]. Leukostasis may cause capillary closure, endothelial death and, ultimately, breakdown of the blood-retina barrier (BRB). Activation of both vascular endothelial cells and circulating immune cells is essential for the increased leukostasis in diabetes [11]. Early work in rodent models of DR indicated that neutrophils and monocytes are key immune cells involved in retinal leukostasis [11–13], and adhesion molecules such as leukocyte function-associated antigen-1 (LFA-1) and vascular cell adhesion molecule 1 (VCAM-1) play an important role in this process [14]. Serra et al. showed that CD11b^+^ bone marrow-derived monocytes from diabetic mice expressed higher levels of CCR5 [11], a chemokine receptor for which its polymorphisms have been associated with diabetes [15]. Clinical studies in patients with type 1 diabetes (T1D) have supported this finding, in which monocytes from T1D patients are activated [16], express higher levels of adhesion molecules such as integrins CD11a and CD11b [17, 18] and have greater potency to bind to endothelial cells [18].

This study was undertaken to uncover the molecular mechanism(s) underlying diabetes-induced leukocyte activation. The results suggest that the expression of phosphorylated signal transducer and activator of transcription (pSTAT3) is increased in leukocytes from T1D patients with early stages of DR and in the Streptozotocin-induced rodent model of T1D. Deletion of the negative regulator of STAT3, the suppressor of cytokine signalling 3 (SOCS3) in myeloid cells, resulted in increased pSTAT3 expression and uncontrolled diabetes-induced leukocyte activation, increased leukostasis and exaggerated retinal vascular degeneration.

## Methods

### Human participants

This study was approved by the Office for Research Ethics Committees Northern Ireland (ORECNI, Ref 14/NI/0084); all study procedures were performed in accordance with the Declaration of Helsinki on research involving human volunteers. Participants were recruited from the eye clinics in the Belfast Health and Social Care Trust, Northern Ireland, UK. Written informed consent was obtained from all participants prior to the undertaking of any study procedure.

The study was designed as a cross-sectional study of T1D patients with DR. Participants were included in the study if they were 18 years of age or older, had been diagnosed of having T1D and if they had either mild NPDR or PDR (active or previously treated and inactive). Exclusion criteria included lack of DR; NPDR more than mild or less than PDR; history of severe cardiac diseases, malignancy within the past 5 years, infectious/non-infectious inflammatory diseases within the previous 2 months; presence of active autoimmune disease; history or concurrent use of immunosuppressive medications or steroids; pregnancy; kidney failure and/or media opacities that would prevent adequate fundus examination. All participants underwent a full ophthalmic examination, including detailed slit-lamp biomicroscopy and confirmation and grading of the presence of mild NPDR or PDR. Twenty millilitre whole blood was drawn into EDTA-treated blood collection tube for evaluating STAT3 activation.

### Experimental animal studies

#### Mice and induction of diabetes

C57BL/6 J WT mice, SOCS3^fl/fl^ and LysM^Cre/+^SOCS3^fl/fl^ mice (all in C57BL/6J background) were bred and maintained at the Biological Resource Unit of Queen’s University Belfast. All animals had free access to food and water and were housed in a temperature- and light-controlled environment with 12 h light/dark cycle. All procedures were approved by the Ethics Committee of Queen’s University Belfast, and complied with the Home Office Animal (Scientific Procedures) Act (UK) and the ARVO (Association for Research in Vision and Ophthalmology) statement for the use of animals in ophthalmic and vision research. Diabetes was induced in 12-week male mice by administrating five daily i.p. injections of Streptozotocin (STZ, Sigma-Aldrich, Dorset UK) (50 mg/kg body weight) [19]. Non-fasting glucose levels were tested 1 week after the animals were rendered diabetic using glucometry (FreeStyle Lite, Abbott laboratories, Dublin, Ireland); animals with glucose concentrations > 13 mmol/l were considered to have developed diabetes. Body weight and non-fasting glucose levels were monitored biweekly. HbA1c levels were determined using Glyco-Tek Affinity Column (Helena Biosciences Europe, Gateshead, UK) when mice were sacrificed.

#### Whole blood RNA extraction and qPCR

Up to 50 μl of mouse whole blood was collected from tail vein and added to RNAprotect blood tube (Qiagen, Manchester, UK) immediately. RNA extraction from whole blood was performed using RNeasy Protect Animal blood kit (Qiagen) following the manufacturer’s instruction. RNA quantity was measured using NanoDrop 1000 Spectrophotometer (Thermo Scientific, Leicestershire, UK). The same amount of RNA from each sample was reverse-transcribed to cDNA using First Strand transcriptase (Invitrogen, Paisley, UK) with random primers following manufacturer’s instruction. Real-time qPCR was performed using Roche SYBR Green Master Mix (Roche, Basel, Switzerland) on Roche LightCycler 480 (Roche). Primers to detect socs3 (forward: cctttgacaagcggactctc, Reverse: gccagcataaaaacccttca) and 18S (aggggagagcgggtaagaga, reverse: ggacaggactaggcggaaca) were designed using primer3 and synthesised by Integrated DNA Technologies (Leuven, Belgium). Data were analysed using delta delta Ct method after normalisation using housekeeping gene 18S.

#### Immunofluorescence staining

Whole mouse eyes were collected and fixed in 2% PFA/PBS for 2 h before transferring to PBS and stored in 4 °C for further processing [20]. For retinal flatmount staining, retinal tissues were permeabilised and blocked in 1% BSA/PBS for 2 h and then incubated in primary antibodies (Biotinylated Griffonia Simplicifolia Lectin I Isolectin B4: 1:50, Vector laboratory, Peterborough, UK; rabbit anti-mouse Collagen IV: 1:50, AbD Serotec®, Kidlington, UK) overnight. After thorough washes, samples were incubated with secondary antibodies (Streptavidin FITC or goat anti-rabbit Alex Fluor 569, all from Invitrogen, UK, 1:200) at room temperature for 2 h. Tissues were flatmounted on glass slides with mounting medium and evaluated under confocal microscope (EZ-C1 confocal system, Nikon UK Limited, Surrey, UK).

#### Leukostasis assay

Leukostasis was evaluated using a protocol published previously with slight modification [19]. Briefly, mice were intravenously injected with 120 μl FITC labelled Concanavalin A (Con A, Vector Lab, UK). After 20 min, mice were deeply anaesthetised with pentobarbital and perfused with PBS under controlled pressure. Eyes were then collected and fixed in 2% PFA for 2 h. Retinas were dissected and flatmounted for confocal microscopy. Adherent Con A^+^ cells in retinal blood vessels were counted manually.

### Flow cytometric analysis in human and mice blood samples

One hundred microliter of human whole blood and 50 μl of mouse blood incubated with/without 100 ng/ml of IL-6 (20 min for human cells, 40 min for mouse cells) were stained for pSTAT3 (Tyr705-FITC from BD biosciences, oxford, UK for human; Tyr705-APC from eBioscience for mouse) following the manufacturers’ instruction. Mouse IgG2a-FITC isotype control (BD Biosciences) or mouse IgG2b Kappa Isotype control (eBioscience) were used. Stained cells were examined by FACS Canto II (BD Biosciences) and data were analysed using the FlowJo software (Version 7, Tree Star, Inc., Ashland, OR, USA).

Thirty microliter of mouse blood were collected through tail-vein prick to EDTA-treated tubes and incubated with antibodies against leukocyte surface markers including CD3, CD19, GR-1, CD11b, LFA-1 (1:100, BD Biosciences), CD62L, (1:100, BioLegend, San Diego, USA) and MHC II (1:100, eBioscience) for 30 min. Red blood cells were then removed with lysis buffer (BD Biosciences) and samples analysed by FACS Canto II (BD Biosciences), and data were analysed using FlowJo (Version 7, Tree Star).

Plasma were collected from control and diabetic mice (with 3 months diabetes duration). Cytokines IL-6, IL-10, TNFα, IFNγ, IL-1β and IL12/IL23p40 as well as chemokine CCL2 were measured by Cytometric Bead Array (CBA; flex set, BD Biosciences) following manufacturer’s instruction. Samples were analysed by FACS Canto II (BD Biosciences) and data were processed by using FCAP array software (BD Biosciences).

### Data analysis

Data are expressed as means ± standard error of the mean (SEM). Graphs and statistical analyses were performed using GraphPad Prism 5 (Graphpad, San Diego, CA, USA). Unpaired *t* test was used to compare two groups; one-way ANOVA with Tukey’s post-hoc test was used to compare three or more groups. Two-way ANOVA was used to compare studies having four groups (SOCS3^fl/fl^ controls, SOCS3^fl/fl^ diabetes, LysM^Cre/+^SOCS3^fl/fl^ controls, LysM^Cre/+^SOCS3^fl/fl^ diabetes). A *p* value of < 0.05 was considered statistically significant. Statistical Package for the Social Sciences, Windows version 24 (SPSS; SPSS Inc., Armonk, NY, USA) was used to carry out linear regression and correlation of duration of diabetes and pSTAT3 expression.

## Results

### Increased expression of pSTAT3 in circulating leukocytes in mNPDR

**Fig. 1:**
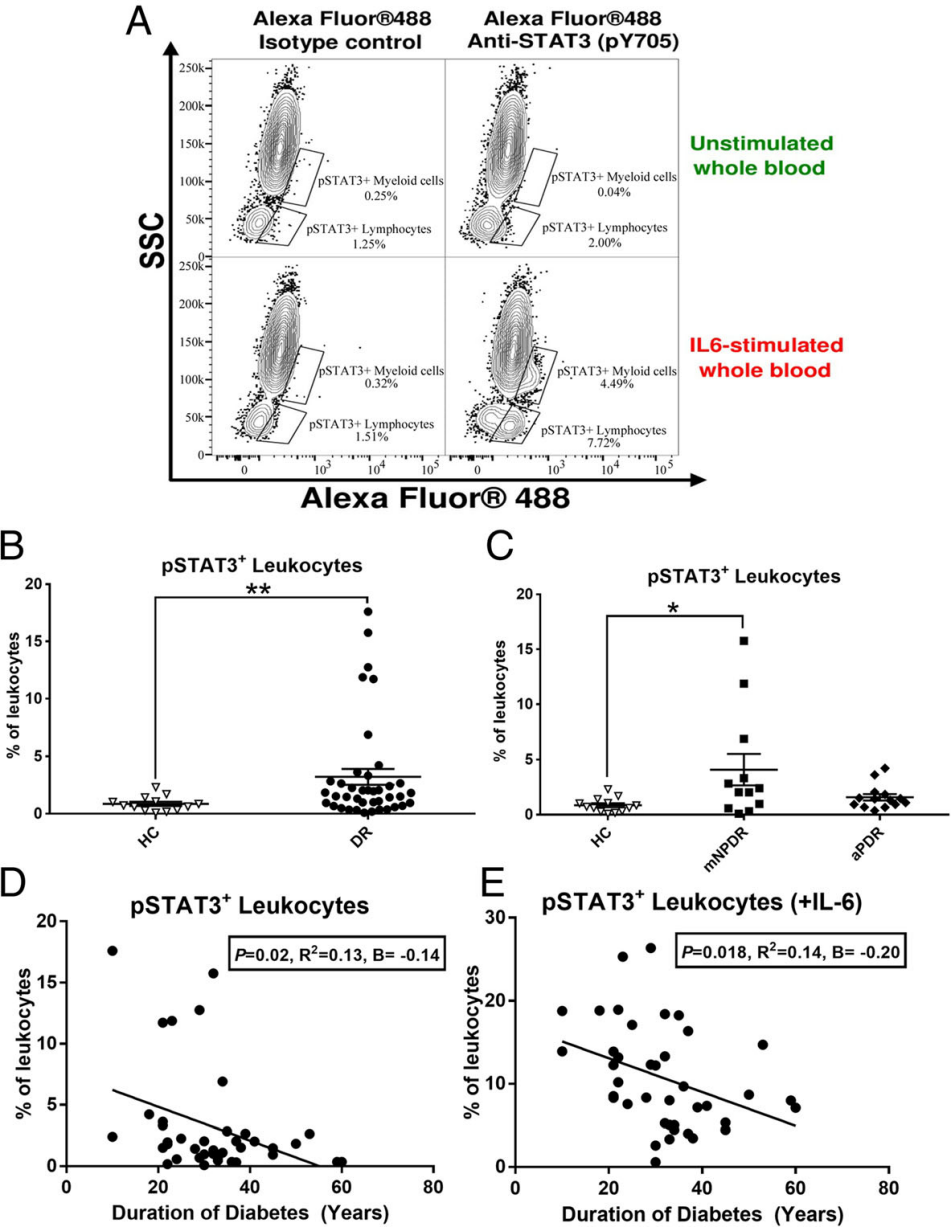
pSTAT3 expression in circulating lekocytes from diabetes patients. **a** Representative image showing gating strategy (Alexa Fluor 488 vs SSC) used to elucidate pSTAT3 expression on peripheral whole blood with/without IL-6 stimulation alongside either Alexa Fluor 488-conjugated Isotype control or anti-STAT3 (pY705) staining. **b** The percentage of total pSTAT3^+^ circulating leukocytes in unstimulated whole blood of all T1D patients (*n* = 39) compared with healthy control (*n* = 13). **c** The percentage of total pSTAT3^+^ circulating leukocytes in unstimulated whole blood of health control (*n* = 13), mNPDR (*n* = 13) and aPDR (*n* = 14). **d**, **e** Correlation of T1D duration against percentages of circulating pSTAT3^+^ leukocytes without IL-6 stimulation (**d**) and with 20 min IL-6 stimulation (**e**). The *B* values (regression coefficient) reported in **d**, **e** are changes in pSTAT3 expression associated with a unit (1 year) increase in T1D duration. The *R*^2^ is the magnitude correlation coefficient between T1D duration and pSTAT3 expression. **b**, **c** Data are presented as mean ± SEM. **b** independent sample *T* test. **c** One-way ANOVA with Tukey’s post-hoc test. *p < 0.05; **p < 0.01. **d**, **e** Linear regression, line on each graph is regression line. *n* = 40

Forty-one T1D patients were recruited into this study with mild non-proliferative diabetic retinopathy (mNPDR) (*n* = 13), active, untreated PDR (aPDR) (*n* = 14) and treated and inactive PDR (iPDR) (*n* = 14). Age- and gender-matched healthy controls (*n* = 13) were also recruited. Characteristics of T1D patients and healthy volunteers are summarised in Additional file 1: Table S1. No statistically significant differences were found between healthy controls and DR patients in demographic factors such as age and gender distribution. There were also no statistically significant differences regarding gender and age between healthy controls and patients with mNPDR or active or inactive PDRs. The expression of pSTAT3 in circulating leukocytes with or without IL6 stimulation was examined by flow cytometry (Fig. 1a). A statistically significantly higher percentage of pSTAT3^+^ leukocytes was observed in T1D patients compared with healthy controls (Fig. 1b). When DR patients were further grouped into mNPDR and aPDR, a statistically significant increased number of pSTAT3^+^ leukocytes was observed in mNPDR, but not aPDR, compared to healthy controls (Fig. 1c).

Diabetes duration is a risk factor for development and progression of DR. A statistically significantly negative correlation between durations of diabetes and pSTAT3 expression [baseline, unstimulated expression (Fig. 1d) and IL-6 stimulation (Fig. 1e)] was found. These results suggest that a higher level of pSTAT3 expression in circulating leukocytes is related to early stages of DR and may contribute to the development of DR.

### Activation of the IL6-STAT3-SOCS3 pathway in circulating leukocytes in diabetic mice

To elucidate the role of the STAT3 pathway in diabetes-induced immune cell activation and its contribution to the initiation of DR, the STZ-induced type 1 diabetic mouse model was used in the rest of the studies.

**Fig. 2:**
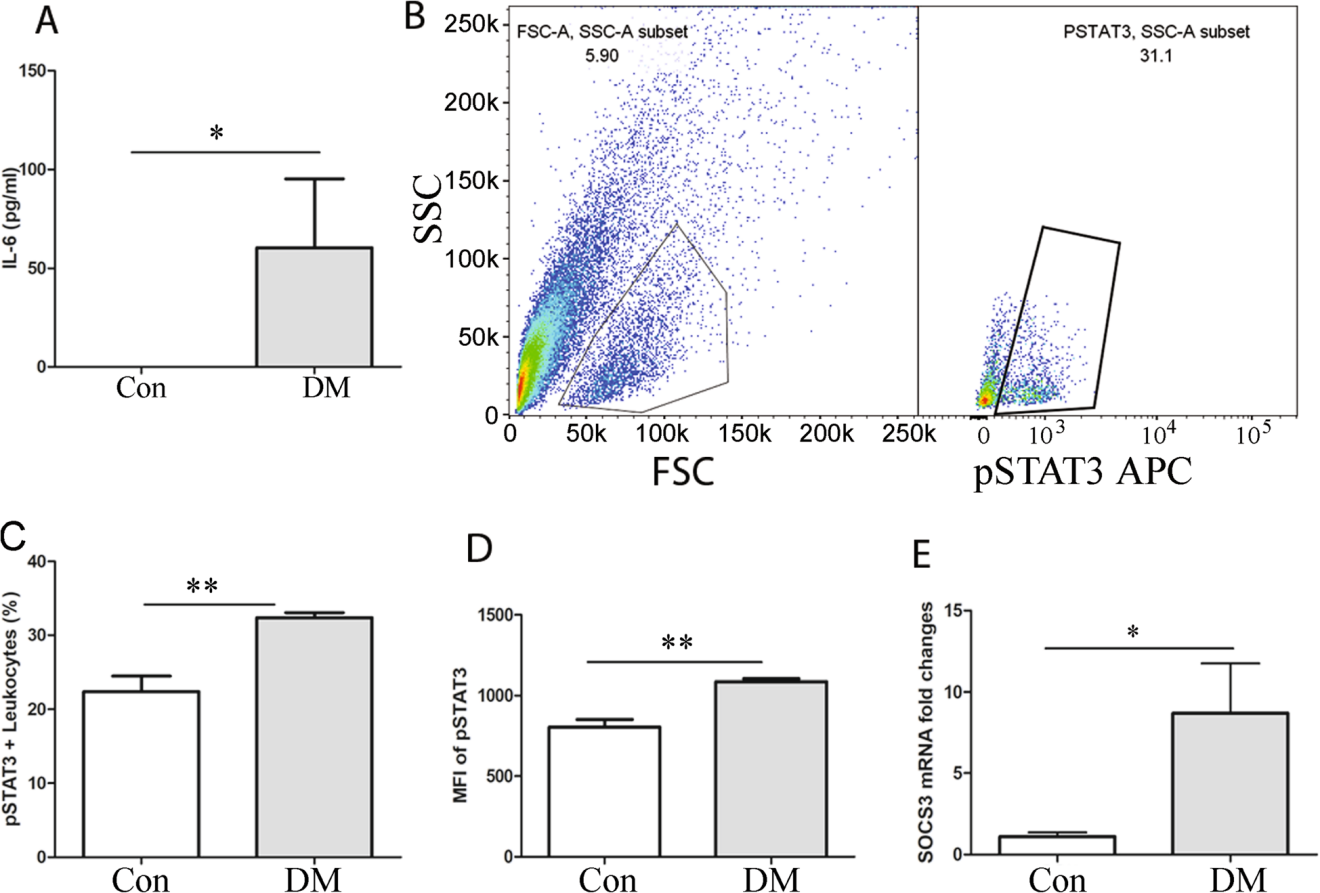
Activation of the IL-6-STAT3-SOCS3 pathway in circulating leukocytes. **a** Plasma cytokine IL-6 in non-diabetic (CON, *n* = 7) and STZ-induced diabetic mice (DM, *n* = 13). **b** The gating strategy to identify pSTAT3^+^ leukocyte by flow cytometry. FSC and SSC were used to identify blood leukocytes. pSTAT3^+^ leukocytes were then identified. **c** The percentage of pSTAT3^+^ leukocytes in non-diabetic (CON, *n* = 5) and diabetic mice (3-month diabetes duration, *n* = 4) after IL-6 stimulation. **d** The mean fluorescent intensity (MFI) of pSTAT3 in circulating leukocytes from control (*n* = 5) and diabetic mice (3-month diabetes duration, *n* = 4) after 40 min IL-6 stimulation. **e** mRNA level of SOCS3 in circulating leukocytes in control (*n* = 4) and diabetic mice (*n* = 8). Data were presented as mean ± SEM. **a** Mann-Whitney test, **c**–**e** Student t test. *p < 0.05; **p < 0.01

CBA assay showed that IL-6 was detected in plasma in seven out of 13 diabetic mice with 3-month diabetic duration, but none in the non-diabetic control mice (*n* = 7 mice) (Fig. 2a, *p* < 0.05, Mann-Whitney test, two tails). IL-10 and other cytokines such as TNFα, IFNγ, IL-1β and IL-12 were below detectable levels (< 12 pg/ml) in both control and diabetic mice. Chemokine CCL2 was not detected in the plasma of healthy control mice, but it was detected between 10~50 pg/ml (22.31 ± 5.18 pg/ml) in diabetic mice (*p* < 0.05, Mann-Whitney test).

STAT3 is activated through phosphorylation of tyrosine 705 (pSTAT3) leading to intranuclear translocation and upregulation of a variety of inflammation/angiogenesis-related genes. Flow cytometry analysis of whole blood after the stimulation of IL-6 showed that the percentage of pSTAT3^+^ leukocytes (Fig. 2b, c) and the geometric mean of fluorescence intensity of pSTAT3 (Fig. 2d) were significantly higher in diabetic mice compared with control non-diabetic mice. SOCS3 is a negative regulator of pSTAT3. Real-time RT-PCR analysis showed that the expression of SOCS3 was increased in circulating leukocytes from diabetic mice compared with non-diabetic controls (Fig. 2e, *p* < 0.05). Our results suggest the activation of the IL6-STAT3-SOCS3 pathway in circulating leukocytes in diabetic mice.

### Activation of circulating immune cells in diabetic mice

**Fig. 3:**
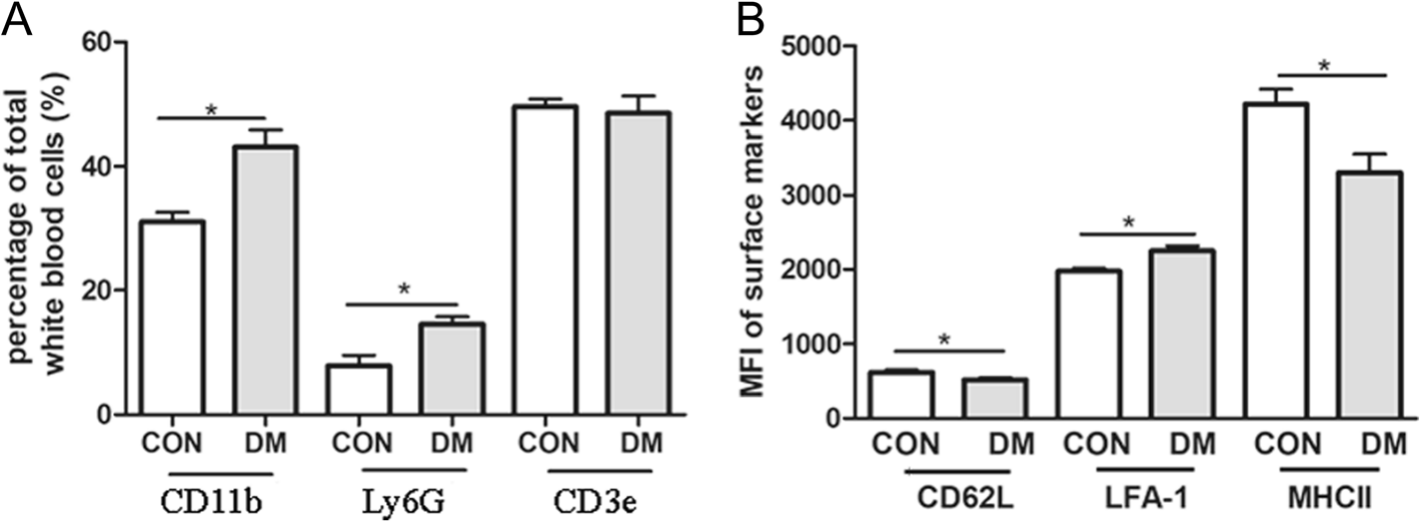
Activation of circulating leukocytes in diabetic mice. **a** FACS analysis shows that CD11b^+^ myeloid cells, Ly6G^+^ neutrophils and CD3^+^ T cells in control (*n* = 4) and diabetic mice (with 3-month diabetes duration, *n* = 5). **b** The mean fluorescence intensity (MFI) of CD62L, LFA-1 and MHC II in circulating CD11b^+^ myeloid cells from healthy control (*n* = 6) and diabetic mice (*n* = 13). Data were presented as mean ± SEM. Student *t* test, * < 0.05

Flow cytometry analysis showed that the percentage of CD11b^+^ or Ly6G^+^ leukocytes of total white blood cells was significantly increased in diabetic mice (Fig. 3a). The percentage of CD3e T cells did not significantly change. Furthermore, the mean fluorescence intensity (MFI) of adhesion molecule LFA-1 was increased, and the expression of l-selectin (CD62L) and MHC-II was decreased, particularly in CD11b^+^ myeloid cells (Fig. 3b). l-selectin is constitutively expressed by all immune cells and the expression is downregulated upon activation. The results suggest that hyperglycaemia increases the population of myeloid-derived cells, induces their activation and upregulates the adhesion molecule expression.

### Diabetes-induced myeloid cell activation is heightened in LysM^Cre/+^SOCS3^fl/fl^ mice

Diabetes-induced leukostasis is known to be caused predominately by myeloid cells, e.g. neutrophils and monocytes [10, 11]. To further understand the role of pSTAT3 in diabetes-induced myeloid cell activation, we used LysM^Cre/+^SOCS3^fl/fl^ mice, in which SOCS3 was deleted in myeloid cells resulting in spontaneous STAT3 activation [21–23]. Under non-diabetic conditions, there was no significant difference between SOCS3^fl/fl^ and LysM^Cre/+^SOCS3^fl/fl^ mice in the distribution of different subsets of circulating leukocytes, including CD3^+^ T cells, CD19^+^ B cells, GR-1^+^ neutrophils, CD11b^+^ myeloid cells and CD56^+^ NK cells [23].

**Fig. 4:**
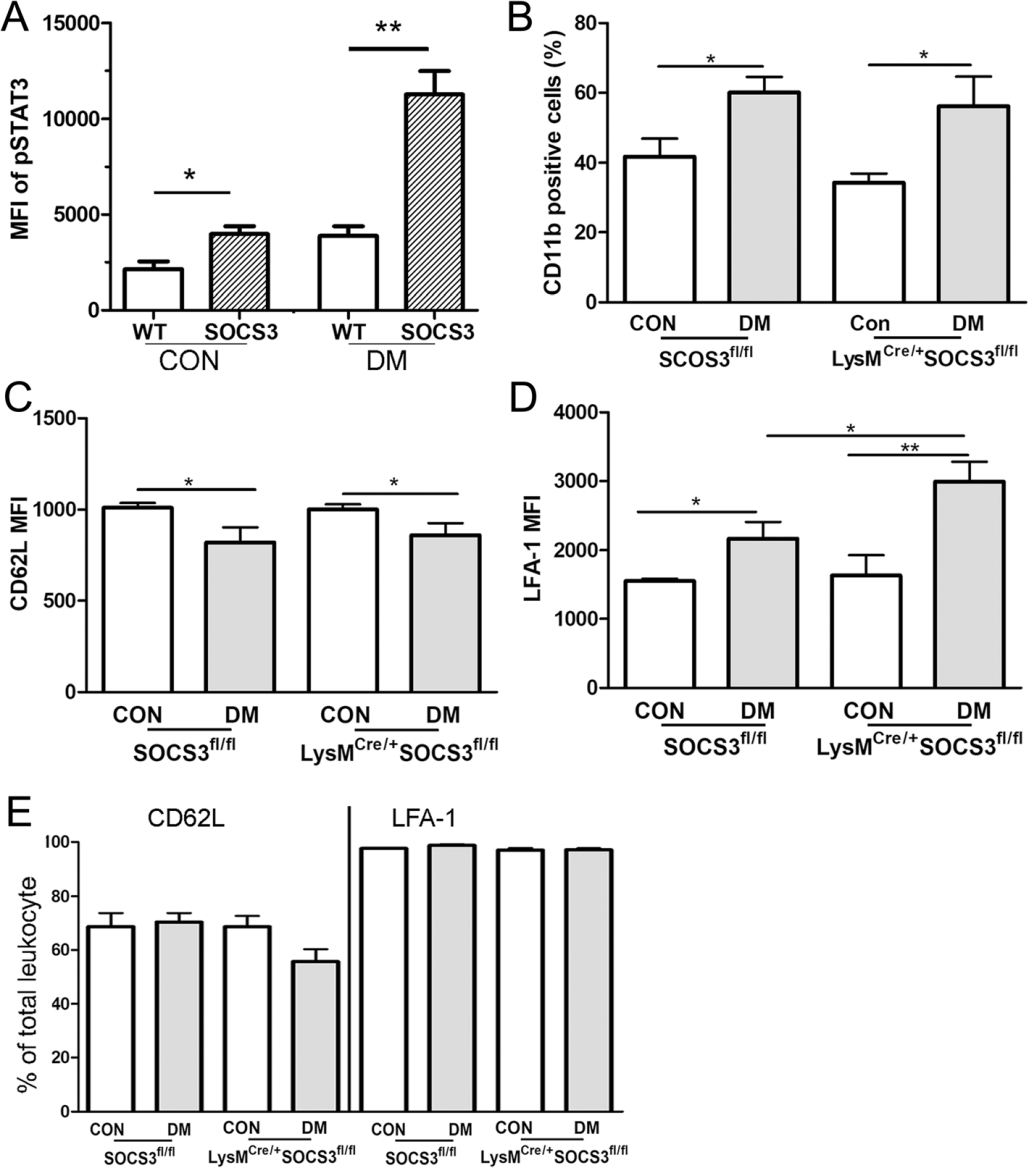
Myeloid cell activation in LysM^Cre/+^SOCS3^fl/fl^ diabetic mice. **a** Fresh blood were collected from WT and LysM^Cre/+^SOCS3^fl/fl^ mice without or with 3-month diabetes and treated with IL-6 for 40 min. pSTAT3 expression was the examined by flow cytometry. Data showing are mean fluorescent intensity (MFI) of pSTAT3 in circulating leukocytes from WT and LysM^Cre/+^SOCS3^fl/fl^ mice with and without diabetes. Student *t* test, *p < 0.05. **b** The percentage of CD11b^+^ cells in SOCS3^fl/fl^ and LysM^Cre/+^ SOCS3^fl/fl^ mice with and without 3-month diabetes. **c**, **d** The mean fluorescent intensity (MFI) of CD62L (**c**) and LFA (**d**) in circulating leukocytes in SOCS3^fl/fl^ and LysM^Cre/+^ SOCS3^fl/fl^ mice with and without diabetes. **e** Percentage of CD62L^+^ and LFA-1^+^ leukocytes in SOCS3^fl/fl^ and LysM^Cre/+^ SOCS3^fl/fl^ mice with and without diabetes. *n* = 4~ 6 per group. Data were presented as mean ± SEM. Two-way ANOVA followed by Bonferroni test. *p < 0.05; **p < 0.01

LysM^Cre/+^SOCS3^fl/fl^ mice and SOCS3^fl/fl^ mice developed comparable levels of diabetes upon STZ injection as they had similar levels of blood glucose (Additional file 1: Figure S1A) and HbA1c (Additional file 1: Figure S1B). Three months after the onset of diabetes, the expression of pSTAT3 was increased in circulating leukocytes and the increment was significantly higher in LysM^Cre/+^SOCS3^fl/fl^ mice compared with SOCS3^fl/fl^ mice (Fig. 4a). The population of CD11b cells was increased at a similar level in SOCS3^fl/fl^ and LysM^Cre/+^SOCS3^fl/fl^ diabetic mice (Fig. 4b). The expression level of CD62L was downregulated (Fig. 4c), whereas the expression level of LFA-1 (Fig. 4d) was upregulated in both SOCS3^fl/fl^ and LysM^Cre/+^SOCS3^fl/fl^ diabetic mice, although the population of CD62L^+^ and LFA-1^+^ cells remained unchanged among all groups (Fig. 4e). The diabetes-induced upregulation of LFA-1 levels was significantly higher in LysM^Cre/+^SOCS3^fl/fl^ mice compared with that in SOCS3^fl/fl^ mice (Fig. 4d). Our results suggest that CD11b^+^ myeloid cells in LysM^Cre/+^SOCS3^fl/fl^ mice were more active than those in SOCS3^fl/fl^ mice under diabetic conditions.

### Deletion of SOCS3 in myeloid cells worsens diabetic retinal vasculopathy

**Fig. 5:**
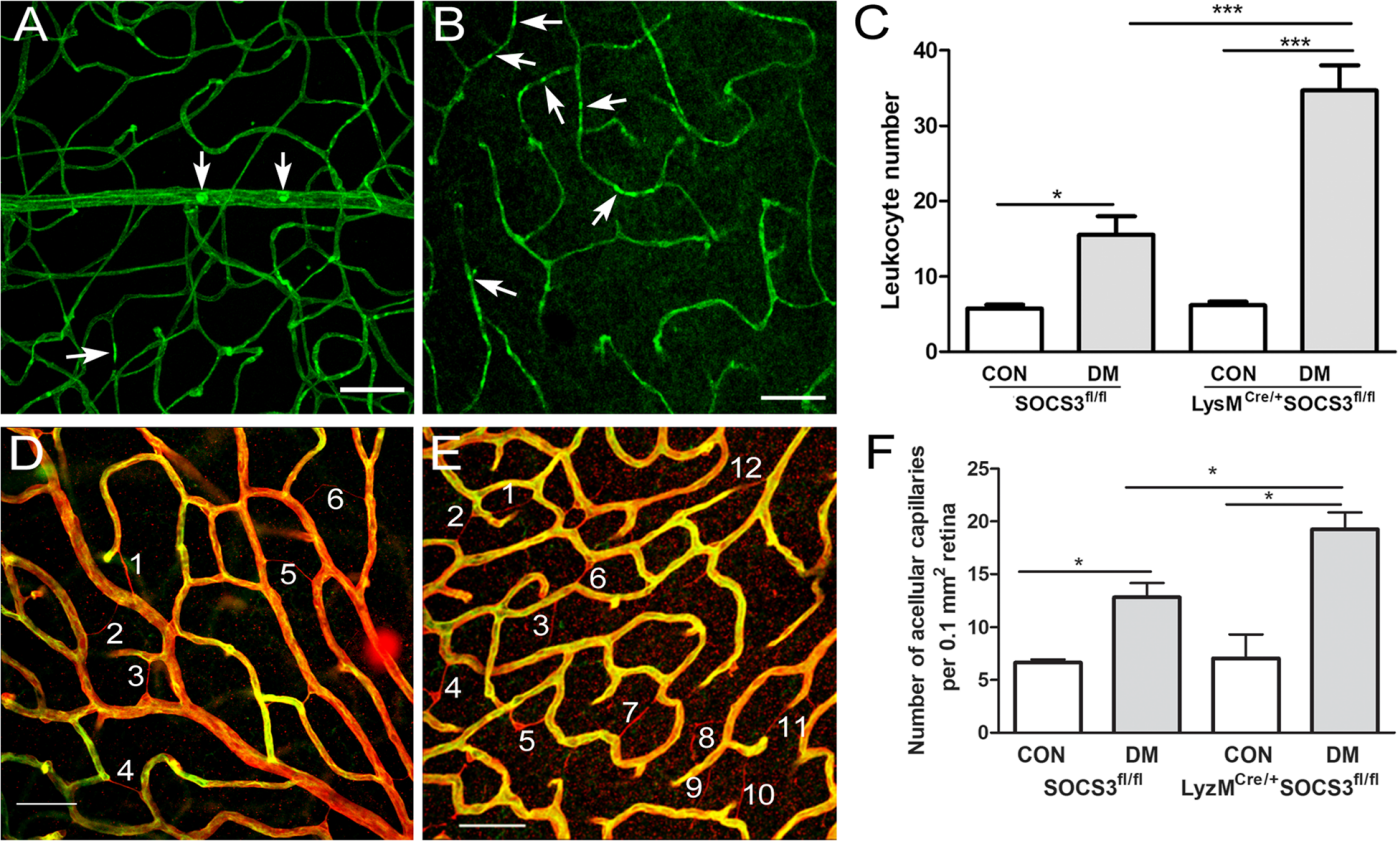
Increased leukostasis and avascular capillaries in LysM^Cre/+^SOCS3^fl/fl^ diabetic mice. **a**–**c** Retinal leukostasis was evaluated by Con-A perfusion in SOCS3^fl/fl^ and LysM^Cre/+^SOCS3^fl/fl^ mice with/without 2-months diabetes and examined by confocal microscopy. **a**, **b** Representative confocal images of retinal flatmount showing entrapped leukocytes (arrows) in diabetic SOCS3^fl/fl^ (**a**) and LysM^Cre/+^ SOCS3^fl/fl^ (**b**). **c** Bar figure showing the number of entrapped leukocytes in SOCS3^fl/fl^ and LysM^Cre/+^SOCS3^fl/fl^ mice. **d**–**f** Acellular capillary was examined by confocal microscopy of collagen IV (red) and Isolectin B4 (green) stained retinal flatmounts from in SOCS3^fl/fl^ and LysM^Cre/+^SOCS3^fl/fl^ mice with/without 6-month diabetes. Representative confocal images of retina flatmount stained with collagen IV (Red) and Isolectin B4 (green) in diabetic SOCS3^fl/fl^ (**d**) and LysM^Cre/+^SOCS3^fl/fl^ (**e**) mice. Acellular capillaries are positive for collagen IV but negative for Isolectin B4 (numbered). **f** Bar figure showing the quantification of acellular capillaries. Data were presented as mean ± SEM. *N* > 6 per group. Two-way ANOVA followed by Bonferroni test. CON: non-diabetes, DM: diabetes. *p < 0.05; ***p < 0.001

To understand whether increased circulating myeloid cell activation in LysM^Cre/+^SOCS3^fl/fl^ diabetic mice leads to more severe diabetic retinal vasculopathy, DR-related vascular changes were investigated in SOCS3^fl/fl^ and LysM^Cre/+^SOCS3^fl/fl^ diabetic mice. Leukostasis was examined by using the Concanavalin A-FITC labelling technique. In healthy mice, 3~ 5 cells/retina were detected and there was no difference between the two strains of mice (Fig. 5a–c). One month after diabetes induction, significantly higher number of Con-A^+^ leukocytes were detected in the retinal vasculature of diabetic mice when compared to that in the non-diabetic counterpart controls (Fig. 5a–c). Furthermore, the number of cells detected in LysM^Cre/+^SOCS3^fl/fl^ diabetic mice (Fig. 5b, c) was significantly higher than that in SOCS3^fl/fl^ diabetic mice (Fig. 5a, c).

The evaluation of acellular capillaries was undertaken using dual staining of Isolectin B4 (for endothelial cells) and collagen IV (for blood vessel basal membrane). Acellular capillaries have basal membrane but no endothelial cells, therefore, are collagen IV positive and Isolectin B4 negative (Fig. 5d–f). A significantly higher number of acellular capillaries were detected in LysM^Cre/+^SOCS3^fl/fl^ diabetic mice than that in the SOCS3^fl/fl^ counterparts (Fig. 5, *p* < 0.05).

## Discussion

Capillary occlusion is an early event of DR and a hallmark of retinal ischemia in patients with diabetes [10]. Entrapment of leukocytes inside retinal blood vessels (leukostasis) is considered as an important contributory factor to vascular damage, at least in experimental models of DR [24–26]. Myeloid-derived cells are the predominant cells in the process of leukostasis [11]. Herein, we showed that pSTAT3 expression in circulating immune cells, including myeloid cells, is upregulated in T1D patients with mNPDR but not aPDR, suggesting that STAT3 activation occurs early in DR and may contribute to the development of this complication. STZ-induced diabetic mice is a model representing early stages of DR (e.g. mild vascular degeneration, BRB leakage but no severe ischemia and angiogenesis). Using this model, we found that the IL-6/STAT3/SOCS3 pathway is activated in circulating immune cells. Furthermore, using the LysM^Cre/+^SOCS3^fl/fl^ mice, where the myeloid cells constitutively express pSTAT3 due to the deletion of its inhibitor SOCS3, we showed that uncontrolled STAT3 activation in myeloid cells leads to more severe diabetes-induced retinal vascular changes. Our results suggest that STAT3-driven immune cell activation plays an important role in early diabetes-induced microvascular degeneration.

Signs of mNPDR include microaneurysms and small haemorrhages or transient BRB leakage [27, 28], which are all related to microvascular damage. Previous studies have demonstrated that STAT3 activation in endothelial cells contributes to the early vascular damage in DR [29–31]. STAT3 may contribute to the early vascular damage through activating genes related to inflammation and angiogenesis such as VEGF and hypoxia-inducible factor 1 [32, 33]. STAT3 activation is also known to be involved in hyperglycaemia-induced endoplasmic reticulum stress in endothelial cells [30]; inhibiting NOX4 can attenuate high-glucose induced reactive oxygen species (ROS) generation and STAT3 activation [29]. Our study suggests that STAT3 activation in leukocytes also contributes to diabetes-induced vascular damage.

How leukocytes are activated in diabetes is not fully understood. Circulating leukocytes originate from bone marrow (BM) haematopoietic stem cells. A previous study has shown that diabetic microenvironment impairs the colony-forming capability of the BM resulting in increased Ly6C^hi^ (pro-inflammatory) circulating monocytes [34]. In this study, the percentage of CD11b^+^ or Ly6G^+^ leukocytes was significantly increased, whereas the percentage of CD3 T cells did not change in the STZ-induced diabetic mice. Further studies will be needed to understand how other leukocytes such as NK cells and B cells are affected by hyperglycemia in this STZ-model. Recently, we have shown that under high glucose conditions, bone marrow-derived macrophages presented a more pro-inflammatory status [35]. It is possible that the high glucose may induce the pro-inflammatory phenotype of macrophages through the STAT3 pathway as STAT3 can be activated by both growth factor receptor and non-receptor tyrosine kinases pathway [36]. Apart from hyperglycaemia, the phenotype and function of circulating cells may also be affected by cytokines, chemokines and other growth factors in the plasma in diabetes. Increased levels of IL-6 and CCL2 have been observed in the plasma [37] as well as in ocular fluids [38, 39] of patients with DR. We have detected increased levels of IL-6 and CCL2 in the plasma from diabetic mice. Neutrophils and monocytes from diabetic patients produced significantly greater amounts of superoxide [16, 40], and are the main sources of pro-inflammatory cytokines [41]. These cytokines and superoxide may affect circulating immune cells and induce pSTAT3 expression [36].

STAT3 is a critical transcription activator in physiological and pathological angiogenesis [42]. Previously, we have shown that STAT3 activation in circulating immune cells contributes to the pathogenesis of neovascular age-related macular degeneration [22]. Interestingly, the expression of pSTAT3 in circulating immune cells was not affected in aPDR patients and more importantly, the expression level was negatively correlated with the duration of diabetes. Macrophages are believed to play an important role in pathogenic blood vessels growth in PDR [43]. Our result suggests that the angiogenic effect of macrophage in PDR is not driven by uncontrolled STAT3 activation in their precursors, i.e. monocytes in circulation. Indeed, late-stage diabetes patients often suffer from impaired wound-healing and reduced angiogenesis. However, our result does not rule out the role of STAT3 in PDR as STAT3 can be activated locally in the ischemic retina. When macrophages or other immune cells migrate to the ischemic retina, they may undergo phenotypic switch and express pSTAT3. Cytokines IL-6, IL-17 and growth factor VEGF and PDGF are potent activators of STAT3, and the vitreous levels of these cytokines/growth factors were significantly higher in PDR patients compared with those in cataract patients [44–46]. The role of pSTAT3 in the pathogenesis of PDR warrants further investigation.

## Conclusions

This study uncovered the novel role of the IL-6/STAT3 pathway in diabetes-induced circulating immune cell activation, and the development of diabetic retinal vasculopathy. Targeting the IL-6/STAT3 pathway in myeloid cells may be a novel approach for the prevention or the treatment of retinal capillary occlusion in DR patients.

## Additional file

Additional file 1: (Patient recruitment criteria, demographic & clinical characteristics of DR patients, and blood glucose/HbA1c levels in LysM^Cre/+^SOCS3^fl/fl^ and SOCS3^fl/fl^ diabetic mice). (DOCX 58 kb)

## Data Availability

The datasets are available from the corresponding authors on reasonable request.

## Abbreviations

aPDR: Active proliferative DR
BRB: Blood-retina barrier
DR: Diabetic retinopathy
iPDR: Inactive proliferative DR
mNPDR: Mild non-proliferative diabetic retinopathy
SOCS3: The suppressor of cytokine signalling 3
STAT3: Signal transducer and activator of transcription 3
STZ: Streptozotocin
T1D: Type 1 diabetes
