# Supplementary material for: STAT3 activation in circulating myeloid-derived cells contributes to retinal microvascular dysfunction in diabetes
Source: J Neuroinflammation. 2019 Jul 8;16:138. doi: 10.1186/s12974-019-1533-1 (PMC6615157; doi:10.1186/s12974-019-1533-1)
Supplement: Supplementary file 1 — (Patient recruitment criteria, demographic & clinical characteristics of DR patients, and blood glucose/HbA1c levels in LysMCre/+SOCS3fl/fl and SOCS3fl/fl diabetic mice). (DOCX 58 kb) [file 12974_2019_1533_MOESM1_ESM.docx]

**Additional file 1**

**Material and methods: Human participant recruitment criteria:**

Case report form (CRF) were used to record information including: medical history ( blood pressure, diabetes status and duration, use of insulin, known evidence of macrovascular or microvascular diseases, previous or ongoing medications), smoking habits (smoker, non-smoker, ex-smoker, unknown or not reported), body weight, height, body mass index (BMI), family history of diabetes. Participants with history of severe cardiac diseases and malignancy within 5 years, infectious or non-infectious inflammatory disease within 2 months, active autoimmune disease, history or current use of immunosuppressive medications, pregnancy, kidney failure, presence of other eye conditions that may affect DR diagnosis, inability to undertake imaging studies were excluded from this study. The diagnosis of DR was confirmed by clinical examination of the fundus.

Table S1 Demographic and clinical characteristics of DR patients and controls.

| Participants characteristics | | All  (n=54; 100%) | Healthy controls (n=13; 24.1%) | DR  (n=41; 75.9%) | DR Subtypes | | *P* value |  |
| --- | --- | --- | --- | --- | --- | --- | --- | --- |
|  |  |  |  |  | Mild  NPDR;  mNPDR  (n=13;  24.1%) | Active PDR; aPDR (n=14; 25.9%) | HC  vs  DR | |
| Age (mean±SD), years | | 47±11.7 | 44.5±9.6 | 47.8±12.27 | 47.8±9.2 | 53.6±12.4 | 0.385^*^ | |
| Female (number (%)) | | 16 (29.6) | 4 (30.8) | 12 (22.2) | 4 (30.8) | 3 (21.4) | 0.918^†^ | |
| Duration of T1D (mean±SD), years | | 24.1±16.8 | N/A | 31.75±11.25 | 28.9±8 | 35.9±11.2 | N/A | |
|  | ^*^ Independent samples t-test; ^†^ Pearson’s chi-square test; *SD* Standard deviation; N/A Not applicable. | | | | | | |  |





Figure S1 LysM^Cre/+^SOCS3^fl/fl^ mice and SOCS3^fl/fl^ mice developed similar level of hyperglycemia after STZ induction. (A), blood glucose levels at different times after STZ injection. (B), HbA1c in LysM^Cre/+^SOCS3^fl/fl^ and SOCS3^fl/fl^ mice without or with 6-month diabetes. n = 8 Means ± SEM, **p<0.01. There is no difference in the levels of HbA1c between diabetic LysM^Cre/+^SOCS3^fl/fl^ mice and diabetic SOCS3^fl/fl^ mice.
